# In the Eye of Transformer: Global–Local Correlation for Egocentric Gaze Estimation and Beyond

**DOI:** 10.1007/s11263-023-01879-7

**Published:** 2023-10-18

**Authors:** Bolin Lai, Miao Liu, Fiona Ryan, James M. Rehg

**Affiliations:** 1 Georgia Institute of Technology, Atlanta, GA 30308 USA; 2 Meta AI, Menlo Park, CA 94025 USA

**Keywords:** Egocentric gaze estimation, Vision transformer, Global–local correlation

## Abstract

Predicting human’s gaze from egocentric videos serves as a critical role for human intention understanding in daily activities. In this paper, we present the first transformer-based model to address the challenging problem of egocentric gaze estimation. We observe that the connection between the global scene context and local visual information is vital for localizing the gaze fixation from egocentric video frames. To this end, we design the transformer encoder to embed the global context as one additional visual token and further propose a novel global–local correlation module to explicitly model the correlation of the global token and each local token. We validate our model on two egocentric video datasets – EGTEA Gaze + and Ego4D. Our detailed ablation studies demonstrate the benefits of our method. In addition, our approach exceeds the previous state-of-the-art model by a large margin. We also apply our model to a novel gaze saccade/fixation prediction task and the traditional action recognition problem. The consistent gains suggest the strong generalization capability of our model. We also provide additional visualizations to support our claim that global–local correlation serves a key representation for predicting gaze fixation from egocentric videos. More details can be found in our website (https://bolinlai.github.io/GLC-EgoGazeEst).

## Introduction

**Fig. 1:**
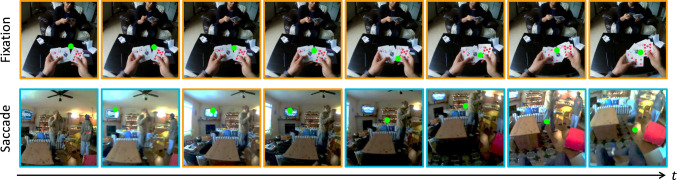
Problem settings of egocentric gaze estimation and gaze saccade/fixation prediction. Given a sequence of video frames, the goal of gaze estimation is to predict where the camera wearer is looking at in each frame. The green dots represent the gaze ground truth (from a wearable eye tracker). In terms of gaze saccade/fixation prediction, the goal is to predict whether a saccade happens within the given input video. In the showing example, frames with blue edges suggest large movements (saccade), while frames with orange edges suggest gaze fixation or subtle gaze movements

Recent findings in cognitive science have validated the capability of eye movements in reflecting the cognitive processes of human (Yarbus, 2013), which are essential for understanding human intention, modeling interactions across a group of people as well as reasoning daily activities in various scenarios (Hayhoe & Ballard, 2005). Recently, more attention has been paid to egocentric gaze behavior modeling (Huang et al., 2018; Li et al., 2021; Huang et al., 2020; Liu et al., 2020; Thakur et al., 2021; Zhang et al., 2018). Such an understanding of visual attention and intention from the first-person perspective can be valuable for many applications, including Augmented Reality (AR), Virtual Reality (VR), and Human-Robot Interaction (HRI). However, how to measure human’s gaze remains a key challenge in this field.

While wearable eye trackers are a standard way to obtain measurements of gaze behavior, they require calibration, consume significant power, and add substantial cost and complexity to wearable platforms. Alternatively, prior works (Li et al., 2013, 2021; Huang et al., 2018; Soo Park & Shi, 2015; Huang et al., 2020; Tavakoli et al., 2019; Thakur et al., 2021; Al-Naser et al., 2019; Huang et al., 2020; Naas et al., 2020) seek to estimate the visual attention of the camera wearer from videos captured from a first-person perspective. In this paper, we address this challenging task of egocentric gaze estimation. Moreover, we introduce a novel task of predicting whether there is gaze saccade within the given egocentric videos. This novel task serves as a key step for understanding human gaze variation and may promise more power-efficient AR user experience. The problem setting of egocentric gaze estimation and egocentric gaze saccade/fixation prediction are introduced in Fig. 1.

**Fig. 2:**
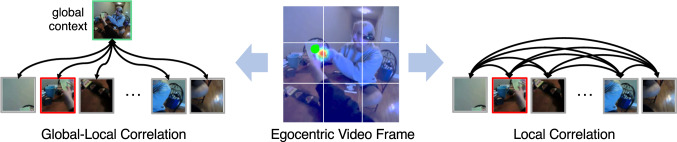
Example of local correlation and global–local correlation for the task of egocentric gaze estimation. The green dot represents the gaze ground truth (from a wearable eye tracker) and the image patch that contains the gaze target has red edges. The heatmap overlaied on the video frame demonstrates the prediction result from our model. Global–local correlation models the connections between the global context and each local patch, making it possible to understand the scene in a holistic perspective, e.g., the camera wearer and social partner are pointing at the salient object. In contrast, local-local correlations may not yield an effective representation of the scene context

The key challenge in modeling gaze behavior from egocentric videos is to effectively integrate multiple gaze cues into a *holistic* analysis of visual attention. Cues include the likelihood that different scene objects are gaze targets (i.e. salience), the relative location of gaze targets within the video frame (i.e. center prior), and the patterns of camera movement that are reflective of visual attention (i.e. head motions accompanying a gaze shift). Prior works on visual saliency prediction propose to use two-stream networks (Wang , 2015), dilated convolutional layers (Yang et al., 2019) or pyramid architectures (Hussain et al., 2022) to enlarge the receptive field, yet incorporating a global representation of the input is still missing from the model designs. Recently, the transformer architecture has achieved great success in various vision tasks by modeling the spatio-temporal correlation among local visual tokens (Strudel et al., 2021; Lou et al., 2021; Liu et al., 2021; Fang et al., 2021; Ren et al., 2022; Lee et al., 2022; Patrick et al., 2021; Ma et al., 2022; Li et al., 2022). Vision transformer shows the potential to effectively capture the global representation, since its receptive field can cover the entire input space. However, the pairwise comparisons performed by standard Self-Attention (SA) mechanism is not optimized for interpreting local video features in the context of the global scene. Figure 2 presents the key role of comparisons between local patches and global context - the gaze target is a salient object pointed at by both the camera wearer and another person. Such a salient object can not be easily localized by only modeling the correlation of local patches.

To this end, our paper introduces a novel transformer-based deep model that explicitly embeds global context and calculates spatio-temporal global–local correlation for egocentric gaze estimation. Specifically, we design a transformer encoder that adopts a global visual token embedding strategy to incorporate the global scene context. The single global visual token is handled together with all local visual tokens by standard self-attention layers in the encoder. We then come up with a novel Global–Local Correlation (GLC) module that highlights the connection between global and local visual tokens by masking out all correlations across local tokens. Finally, we adopt a transformer-based decoder to produce gaze prediction output. To the best of our knowledge, this is the first work applying vision transformer to egocentric gaze estimation. As shown in the heatmap from Fig. 2, our model understands the scene in a holistic view and successfully captures the gaze target with the proposed global context embedding and global–local correlation module.

We exhaustively evaluate our approach on two egocentric video datasets – EGTEA Gaze+ (Li et al., 2018) and Ego4D (Grauman et al., 2022). To begin with, we investigate different strategies for global context embedding and show the contribution of involving the global token and the global–local correlation module. We then compare with all prior works of egocentric gaze estimation on the two datasets. We also apply our model to the novel saccade/fixation prediction problem, and traditional action recognition problem to demonstrate the generalization capability of our model. Our proposed model is easy to incorporate into existing transformer-based video analysis architectures, so we implement all experiments with two different transformer backbones. Our method improves the performance of both backbones and finally yields an *improvement of +3.9% in F1 score* over the previous state-of-the-art method for egocentric gaze estimation. It also boosts the performance on egocentric action recognition and gaze saccade/fixation prediction tasks by a notbale margin. The codes and pretrained models are publicly available to the research community. In summary, this work makes the following contributions:

- We introduce the first transformer-based approach to address the challenging task of egocentric gaze estimation, and introduce a novel task of gaze saccade/fixation prediction from egocentric video.
- We utilize a global visual token embedding strategy to incorporate global visual context into self-attention, and further introduce a novel Global–Local Correlation module to explicitly model the correlation between global context and each local visual token.
- Our novel design obtains consistent improvement on the EGTEA Gaze+ (Li et al., 2018) and Ego4D (Grauman et al., 2022) datasets and outperforms the previous state-of-the-art method by +3.9% on EGTEA and +5.6% on Ego4D in terms of F1 score. Importantly, this is the first work that uses the Ego4D dataset for egocentric gaze estimation, which serves as important benchmark for future research in this direction.
- We provide more insights of our model by applying it to saccade/fixation prediction and egocentric action recognition tasks. We also visualize correlation weights to show more evidence of the global–local correlation mechanism.

An early version of this paper (Lai et al., 2022) was accepted by BMVC 2022 and then invited this special issue. This paper further extends our previous conference version in several important aspects. First, we introduce a novel task of recognizing saccade gaze movements from the egocentric videos. Second, we conduct additional experiments using recent MotionFormer backbone (Patrick et al., 2021). Our new results suggest that GLC module can be easily plugged into other transformer-based backbones and can produce consistent performance gain on egocentric gaze estimation. Third, we show our model design can also benefit saccade/fixation prediction performance, suggesting global context also contributes to the understanding of rapid gaze movements. Finally, we provide more visualizations of gaze estimation results and correlation weights in GLC module.

This paper is organized in the following order. Section 2 reviews all related works about egocentric gaze estimation, vision transformer and visual saliency modeling, and highlight the difference between prior works and this paper. Section 3 elaborates details of the proposed model. Section 4 presents implementation details for each experiment and the experimental results. Section 5 lists the current limitation of our model and promising future works. Section 6 summarizes all findings of this paper.

## Related Work

The computational analysis of human gaze behavior is a long-established topic. For example, earlier works consider the problem of eye tracking (Krafka et al., 2016; MacInnes et al., 2018; Ye et al., 2012), which addresses the problem of tracking the gaze movement based on closeup view of human faces or eyes. Moreover, another topic on gaze target prediction(Chong et al., 2020, 2018; Kellnhofer et al., 2019; Nonaka et al., 2022) aims at predicting the gaze target of a subject from the third-person view. In contrast to these prior works, we address the problem of predicting the gaze target directly from egocentric videos captured by wearable cameras. In this paper, we mainly discuss the most relevant prior works on egocentric gaze estimation and related works on transformer-based video representation learning and video saliency prediction.

### Egocentric Gaze Estimation

Previous works focuses on analyzing human daily activities from egocentric videos (Li et al., 2013, 2021; Huang et al., 2018; Liu et al., 2020; Soo Park & Shi, 2015; Huang et al., 2020; Tavakoli et al., 2019; Zhang et al., 2017; Thakur et al., 2021; Al-Naser et al., 2019; Huang et al., 2020; Naas et al., 2020; Jia et al., 2022; Liu et al., 2022). Here, we discuss the most relevant works that develop deep models for egocentric gaze estimation. Zhang et al. (2017) used deep models and an adversarial network to forecast egocentric gaze location in future video frames, which can also be applied to estimate gaze target in current frames by replacing the labels. They further improve this model by adding another branch to incorporate prior information (Zhang et al., 2018). Huang et al. (2018) proposed to explicitly model the temporal attention transition using a LSTM-based architecture and incorporate it into saliency-based models for gaze estimation. Tavakoli et al. (2019) investigated the impact of various factors on egocentric gaze estimation and provided guidance for future work. Another research field is to leverage the relation of human’s action and gaze behavior and model them jointly. Li et al. (2018) sampled a gaze distribution map from the lower layer and used it to selectively pool the features learned by the higher layer. Inspired by this work, Huang et al. (2020) introduced a multi-stream network to enable gaze and action to serve as contexts for each other.

In addition, there exit many works about the variants of egocentric gaze estimation which expand its applications in various scenarios. Soo Park and Shi (2015) introduced the novel problem of predicting joint attention during social interaction using egocentric videos. Huang et al. (2020) collected a new egocentric video dataset and developed a graphical model to detect joint attention. Thakur et al. (2021) proposed a multi-modal network that uses both video and inertial measurement unit data for more accurate egocentric gaze estimation. Naas et al. (2020) developed a tiling scheme for gaze prediction which enables a more efficient VR content delivery.

All of these prior works did not embed global context explicitly or model the connection between local and global visual representations as in our model, which could limit the capability of their models. Additionally, we are the first to develop a transformer-based architecture to address the problem of egocentric gaze estimation.

### Vision Transformer

Transformer architecture is first proposed by Vaswani et al. (2017) and inspires many large language models (Devlin et al., 2018; Liu et al., 2019; Brown et al., 2020). Recently, vision transformers (Dosovitskiy et al., 2022) have demonstrated superior performance on image classification  (Dai et al., 2021; Liu et al., 2021; Wang et al., 2021; Ren et al., 2022; Yang et al., 2021; Lee et al., 2022), detection (Dai et al., 2021; Carion et al., 2020; Dai et al., 2021; Fang et al., 2021), segmentation (Strudel et al., 2021; Wang et al., 2021; Zheng et al., 2021; Cheng et al., 2022; Zhang et al., 2022), saliency prediction (Ma et al., 2022; Lou et al., 2021; Liu et al., 2021) and video analysis (Arnab et al., 2021; Neimark et al., 2021; Patrick et al., 2021; Fan et al., 2021; Li et al., 2022; Bertasius, Wang and Torresani, 2021; Wang & Torresani, 2022; Liu et al., 2022). In this section, we focus on reviewing previous works that use vision transformers for pixel-wise visual prediction and video understanding. More related works of saliency prediction is elaborated in Sect. 2.3.

Strudel et al. (2021) developed the first transformer-based architecture for semantic segmentation. Cheng et al. (2022) further unified semantic, instance, and panoptic segmentation in one transformer architecture. Ma et al. (2022) expanded transformers to visual saliency forecasting by using self-attention to capture the correlation between past and future frames. Liu et al. (2021) built a transformer-based model to detect salient objects on RGB-D images. They fused the embeddings of the two modalities by using query from RGB frames and key and value from depth images.

In terms of video transformer, Bertasius et al. (2021) proposed TimeSformer for video action recognition, which is the first transformer-based architecture for video understanding. A similar idea was also explored by Arnab et al. (2021). They downsampled the resolution of input video segment by multiple steps before feeding it into transformer layers. Fan et al. (2021) designed a multiscale video transformer balancing computational cost and action recognition performance. This architecture was further improved by rearranging the layers in each transformer block (Li et al., 2022). Patrick et al. (2021) proposed the trajectory attention mechanism to track the same object in each video frame. Liu et al. (2022) extended the 2D swin-transformer (Liu et al., 2021) to a 3D architecture for action recognition.

Inspired by these successful applications of transformer architectures, we present the first work that uses a vision transformer to address the challenging task of egocentric gaze estimation. In addition, we introduce the novel Global–Local Correlation (GLC) module that provides additional insight into video representation learning with self-attention. We implement this module on two video transformer backbones (Fan et al., 2021; Patrick et al., 2021) and conduct thorough experiments in this paper.

**Fig. 3:**
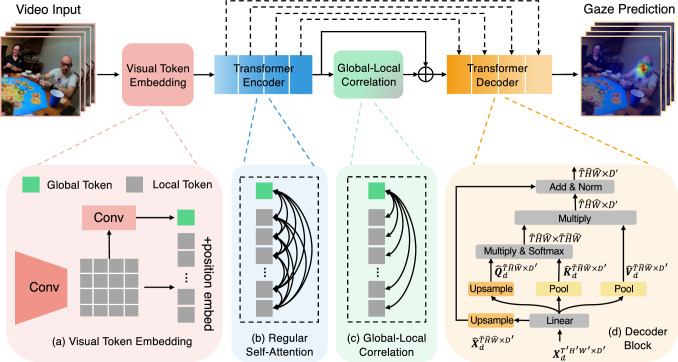
Architecture of the proposed model. The model consists of four modules – **a** Visual Token Embedding Module encodes the input into local tokens and one global token, **b** Transformer Encoder is composed of multiple regular self-attention and linear layers, **c** Global–Local Correlation Module models the correlation of global and local tokens, and **d** Transformer Decoder maps encoded video features from Transformer Encoder and GLC to gaze prediction. $\oplus$ denotes concatenation along the channel dimension

### Visual Saliency

Visual saliency prediction has been well studied in computer vision in recent years (Pan et al., 2017; Wang et al., 2017; Che et al., 2019; Wu et al., 2020; Kroner et al., 2020; Jia & Bruce, 2020; Sun et al., 2022; Lou et al., 2021; Wang et al., 2021; Chen et al., 2021; Khattar et al., 2021; Bellitto et al., 2021; Tsiami et al., 2020; Jiang et al., 2022). Kruthiventi et al. (2017) developed a deep neural network with various kernel sizes to capture saliency features at different scales. Liu et al. (2018) calculated the relation weights between each pixel and the remaining pixels to embed the most relevant contextual features. Zhuge et al. (2022) improved the integrity of detected saliency objects by using integrity channel enhancement mechanism and part-whole verification module. In terms of saliency prediction in videos, Wang et al. (2017) expanded image saliency models to videos by incorporating a new branch to handle temporal information. Wu et al. (2020) proposed SalSAC, which shuffles features of different CNN layers and feeds them to a correlation-based ConvLSTM. Wang et al. (2021) used multiple spatio-temporal self-attention modules to address the limitation of fixed kernel size in 3D models and to model long-range temporal dependencies. Chen et al. (2021) decomposed video saliency prediction into spatial pattern capture and spatio-temporal reasoning. Lou et al. (2021) combined a convolutional network and transformer architecture to model the long-range spatial context. Liu et al. (2023) proposed short-global and long-local attention mechanisms to integrate contexts from neighboring frames.

While visual saliency prediction localizes interesting spatial regions as potential attention targets, egocentric gaze estimation seeks to determine the gaze target of the camera wearers as they interact with a scene. In saliency prediction, cameras typically keep stable and move slowly and salient objects could dominate the view. However, the scene context captured from egocentric video is complex and rapidly changing, which requires a gaze estimation model with the ability of explicitly reasoning about the correlation between local visual features and global scene context. In our experiment section, we demonstrate that our proposed GLC module can significantly benefit gaze estimation performance under this challenging setting.

**Table 1:** Evaluation of different global embedding approaches and global–local correlation module

Methods	EGTEA Gaze+			Ego4D		
	F1	Recall	Precision	F1	Recall	Precision
MViT (Fan et al., 2021)	43.0	57.8	34.2	40.9	57.4	31.7
MViT + (a)	43.4	58.4	34.5	41.5	56.8	32.6
MViT + (b)	43.5	59.2	34.4	41.4	57.3	32.4
MViT + (c)	43.7	58.3	34.9	41.3	57.5	32.2
MViT + (d)	43.9	59.0	34.9	41.7	57.6	32.7
MViT + (d) + SA	44.1	58.8	**35**.**3**	42.1	**58**.**5**	32.9
MViT + (d) + GLC	**44**.**8**	**61**.**2**	**35**.**3**	**43**.**1**	57.0	**34**.**7**

(a)(b)(c)(d) are different global embedding strategies elaborated in Sect. 3.1 and Fig. 4. *SA* and *GLC* denote regular self-attention and global–local correlation module, respectively. Please refer to Sects. 4.2.1 and 4.2.2 for more explanations

## Method

Given an input egocentric video clip with fixed length *T* and spatial dimension $H\times W$, our goal is to predict the gaze location in each video frame. Following Li et al. (2018), we consider the gaze prediction as a probabilistic distribution defined on the 2*D* image plane.

Figure 3 presents an overview of our proposed method. We use the recent multi-scale video transformer (MViT) (Fan et al., 2021) or MotionFormer (Patrick et al., 2021) architecture as the backbone network for video representation learning. We extend the backbone by designing the *Visual Token Embedding Module* to generate the spatio-temporal tokens of both local visual patches and global visual context and feed them into the standard *Multi-Head Self-Attention Module*. We then utilize a novel *Global–Local Correlation (GLC) Module* to explicitly model the correlation between global and local visual tokens for gaze estimation. Finally, we make use of the *Decoder Network* to predict the gaze distribution based on the learned video representation from the GLC module.

### Transformer Encoder with Global Visual Token Embedding

**Visual Token Embedding**. We split the input video sequence into non-overlapping patches with size $s_T\times s_H\times s_W$ and adopt a linear mapping function to project each flattened patch into *D*-dimension vector space. Following MViT (Fan et al., 2021), this is equivalent to a convolutional layer with a stride of $s_T\times s_H\times s_W$ and a number of output channels of *D*. This operation results in *N* tokens where $N=\frac{T}{s_T}\times \frac{H}{s_H}\times \frac{W}{s_W}$. In addition, the learnable positional embedding $\varvec{E}\in \mathbb {R}^{N\times D}$ is added to the local tokens. Our key insight is to further embed global information into a global visual token using convolutional operations, as illustrated in Fig. 3a. Since there is a single global token, it does not require positional embedding.

**Fig. 4:**
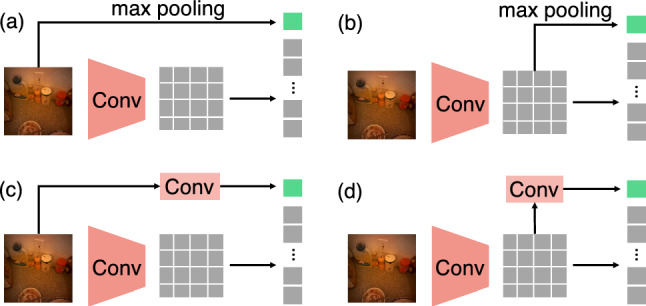
Four different approaches of global visual token embedding

In our experiments, we examine four global visual embedding strategies as demonstrated in Fig. 4. We (a) implement max pooling on input frames directly, and (b) implement max pooling on unflattened local visual tokens. For (c) and (d), we replace max pooling operations in (a) and (b) with a sequence of convolutional layers. Specifically, for global embedding in (d), we use three additional layers to downsample unflattened local tokens to produce a single global token. In (c), input video frames are first fed into a convolutional layer that is identical to the layer used for local token embedding. Then, the output is passed to a sequence of convolutional layers identical to (d). The experimental results of the four strategies are reported in Sect. 4.2.1 and Table 1. The strategy (d) provides the best gaze estimation performance in our experiments and we thereby use (d) in the final version of our model.

**Multi-Head Self-Attention Module**. The *N* local tokens and one global token are fed into a transformer encoder consisting of multiple self-attention blocks. The number of local tokens is downsampled after each self-attention block, while the number of global tokens remains 1. Suppose the input of the *j*-th layer of encoder is $\varvec{X}_e^{(j)}=[\varvec{x}_i^{(j)}]_{i=1}^{N_j+1}\in \mathbb {R}^{(N_j+1)\times D_j}$, where $N_j$ is the number of local tokens, $D_j$ is the vector length of each token and $\varvec{x}_i^{(j)}$ is the *i*-th row of $\varvec{X}_e^{(j)}$ denoting the *i*-th token of size $1\times D_j$. For simplicity, we omit subscript and superscript of *j* and multi-head operations in the following equations. In each self-attention layer, correlations are calculated in each token pair as shown in Fig. 3b. They are used to reweight values of each token after softmax. Formally, we denote the query, key and value matrices of each self-attention layer in an encoder block as $\varvec{Q}_e^{(N+1)\times D}=[\varvec{q}_i]_{i=1}^{N+1}$, $\varvec{K}_e^{(N+1)\times D}=[\varvec{k}_i]_{i=1}^{N+1}$ and $\varvec{V}_e^{(N+1)\times D}=[\varvec{v}_i]_{i=1}^{N+1}$. The self-attention in transformer encoder is formulated as

1 $$\begin{aligned} \begin{aligned}&Attention(\varvec{Q}_e, \varvec{K}_e, \varvec{V}_e) \\ {}&\,= Softmax(\varvec{Q}_e\varvec{K}_e^T / \sqrt{D})\varvec{V}_e \in \mathbb {R}^{(N+1)\times D}. \end{aligned} \end{aligned}$$

Finally, we attach a standard linear layer after the self-attention operation.

### Global–Local Correlation

Even though global information has been explicitly embedded into the global visual token in our model, the transformer encoder treats the global and local tokens equivalently as shown in Eq. 1 and Fig. 3b. In this case, global–local correlation is diluted by correlations among the local tokens, limiting its impact on gaze estimation. In order to address this problem, we propose to increase the available capacity to model global–local token interactions. Our solution is a novel Global-Local Correlation module described in Fig. 3c.

Formally, we denote the global token as the first row vector of $\varvec{X}_e$, $i.e. , \varvec{x}_1$. Thus $\varvec{q}_1$, $\varvec{k}_1$ and $\varvec{v}_1$ are the query, key and value projected from the global token, respectively. To explicitly model the connection between global and local visual features, we only calculate the correlation between each local token and the global token, i.e., $Correlation(\varvec{x}_i, \varvec{x}_1)$, as well as its self-correlation, i.e., $Correlation(\varvec{x}_i, \varvec{x}_i)$. Then correlation scores are normalized by softmax to further re-weight the values. We exploit a suppression matrix (Liu et al., 2021) $\varvec{S}^{(N+1)\times (N+1)}$ to suppress the correlation of other tokens, where

2 $$\begin{aligned} \varvec{S}^{(N+1)\times (N+1)} = [s_{ij}],\quad s_{ij}=\left\{ \begin{array}{ll} 0, &{}\textrm{if}\; i=j \; \textrm{or} \; j=1 \\ \lambda , &{}\textrm{otherwise}. \end{array} \right. \end{aligned}$$

We assign zeros to the diagonal and the first column in $\varvec{S}$ and set a large value $\lambda$ for the other elements. We follow the empirical choice from the implementation of Liu et al. (2021) and set $\lambda =10^8$ in our experiments. Formally, the proposed GLC can be formulated as

3 $$\begin{aligned} \begin{aligned} GLC&(\varvec{Q}_e, \varvec{K}_e, \varvec{V}_e) \\ {}&= Softmax((\varvec{Q}_e\varvec{K}_e^T - \varvec{S}) / \sqrt{D})\varvec{V}_e \in \mathbb {R}^{(N+1)\times D} \end{aligned} \end{aligned}$$

In this way, we keep the values on the first column and the diagonal, and map them into probability distributions, while values in other positions are nearly “masked out” after the softmax. Residual connections and linear layers are also used in the GLC module as in the regular self-attention block. Finally, the output tokens from the GLC are concatenated with those from the transformer encoder in the channel dimension. We denote outputs of the GLC and the last encoder block as $\varvec{X}_e^{GLC}\in \mathbb {R}^{(N+1)\times D}$ and $\varvec{X}_e^{SA}\in \mathbb {R}^{(N+1)\times D}$. The concatenation can then be formulated as $\varvec{X}_e = \varvec{X}_e^{SA} \oplus \varvec{X}_e^{GLC} \in \mathbb {R}^{(N+1)\times 2D}$. The fused tokens $\varvec{X}_e$ are subsequently fed into the transformer decoder for gaze estimation.

### Transformer Decoder

To produce the gaze distribution with the desired spatio-temporal resolution, we adopt a decoder to upsample the encoded features. We utilize a transformer decoder based on the multiscale self-attention block of MViT (Fan et al., 2021). Suppose each decoder layer takes visual features $\varvec{X}_d\in \mathbb {R}^{T'H'W'\times D'}$ as inputs and the corresponding query, key and value matrices are $\varvec{Q}_d^{T'H'W'\times D'}$, $\varvec{K}_d^{T'H'W'\times D'}$ and $\varvec{V}_d^{T'H'W'\times D'}$. As shown in Fig. 3d, we replace the original pooling operation for the query matrix with an upsampling operation implemented with trilinear interpolation and keep the pooling for the key and value matrices. Following Fan et al. (2021), $\widehat{\varvec{Q}}_d$ is obtained by applying a deconvolutional operation on $\varvec{Q}_d$, while $\widehat{\varvec{K}}_d$ and $\widehat{\varvec{V}}_d$ are obtained by applying convolutional operations on $\varvec{K}_d$ and $\varvec{V}_d$. Then, the output of self-attention is calculated in the same way as Eq. 1. In addition, we keep the skip connection in the self-attention layers and replace the pooling operation in skip connections with trilinear interpolation, which produces the upsampled output with dimension $\widehat{T}\widehat{H}\widehat{W}\times D'$. Our decoder is composed of 4 decoding blocks. Skip connections are used to combine intermediate features of the encoder with corresponding decoder features. Finally, another linear mapping function is used to output the final gaze prediction.

### Network Architecture and Model Training

We adopt MViT (Fan et al., 2021) and MotionFormer (Patrick et al., 2021) as the backbones, with weights initialized from Kinetics-400 pretraining (Kay et al., 2017). The GLC module and decoder are initialized with Xavier initialization (Glorot and Bengio, 2010). For MViT, the token embedding stride is set as $s_T=2$, $s_H=4$ and $s_W=4$ and the embedding dimension is $D=96$. The encoder is composed of 16 self-attention layers that are divided into 4 blocks. The number of tokens is downsampled at the transition between two blocks. For MotionFormer, the token embedding stride is set as $s_T=2$, $s_H=16$, $s_W=16$ and the embedding dimension is $D=768$. The encoder consists of 12 layers with trajectory self-attention. The number of tokens doesn’t change in the encoder. We build the decoder with 4 decoder blocks corresponding to the 4 blocks in the encoder. After getting raw output from decoder, softmax is applied on each frame with a temperature $\tau$. This can be formally written as $\hat{p}_{ij} = \frac{\exp (\hat{y}_{ij} / \tau )}{\sum _{i,j}\exp ({\hat{y}_{ij} / \tau )}}$ where $\hat{y}_{ij}$ is the logit at location (*i*, *j*) from the model and $\hat{p}_{ij}$ is probability after softmax. In experiments, $\tau$ is empirically set as 2. We use KL-divergence loss to capture the difference between labels and predictions. The model is trained using AdamW (Loshchilov & Hutter, xxxx) optimizer with a batch size of 16. We adopt a warm-up training strategy that increases learning rate from $10^{-6}$ to $10^{-4}$. Then the learning rate decreases in compliance with cosine annealing scheme (Loshchilov & Hutter, 2016).

## Experiment

In this section, we show the experimental setup and detailed results. We first elaborate the two datasets used in our experiments, evaluation metrics and data processing details. Second we show exhaustive ablation studies for egocentric gaze estimation and compare with prior works. Third, we validate the generalization capability of our model by applying it to gaze saccade/fixation prediction and egocentric action recognition. Finally, we visualize the predictions and correlation weights in GLC module to provide more insights.

### Datasets and Metrics

**Datasets.** We conducted our experiments on two egocentric video datasets with gaze tracking data serving as ground truth – EGTEA Gaze+ (Li et al., 2018) and Ego4D (Grauman et al., 2022). The EGTEA Gaze+ dataset is captured under the meal preparation setting, which involves a great deal of hand-object interactions. We used the first train/test split from EGTEA Gaze+ in our experiments (8299 clips for training and 2022 clips for testing). The Ego4D dataset includes 27 videos of 80 participants totaling 31 h with gaze tracking data captured under the social setting. We split the long videos into 5-second video clips and pick clips containing gaze fixation. We used 20 videos (15,310 clips) for training and the other 7 videos (5202 clips) for testing. Note that we keep using the same train/test split for all the three tasks – egocentric gaze estimation, gaze saccade/fixation prediction and action recognition. Importantly, this is the first work that uses the Ego4D dataset for egocentric gaze estimation, and we have made our split publicly available to drive future research on this topic.

**Evaluation Metrics.** Following Li et al. (2018, 2021) (the source of the EGTEA Gaze+ dataset), we adopt F1 score, recall, and precision as the evaluation metrics for gaze estimation. Note that we do not consider AUC score as our main metrics, since AUC performance can become saturated due to the long-tailed nature of the distribution of gaze in a single frame. In terms of saccade/fixation prediction, we primarily measure the performance by average F1 (average of F1 scores of the two categories) and mean class average (following Li et al. (2021)) because of the imbalance of saccade and fixation, but regular accuracy metric is also provided for reference. For action recognition, we directly follow prior works (Li et al., 2021; Hao et al., 2022) and adopt top-1 accuracy, top-5 accuracy and mean class accuracy.

**Data Processing.** At training time of egocentric gaze estimation, we randomly sample 8 frames from each video with a sampling interval of 8 as input (i.e. selecting 8 frames from a 72-frame window with equal spacing). All videos are spatially downsampled to 256 in height while keeping the original aspect ratio. We further implement multiple data augmentations including random flipping, shifting, and resizing. We then randomly crop each frame to get an input with dimensions $8\times 256\times 256$. The output from the decoder is a downsampled heatmap with dimension $8\times 56\times 56$. For visualization, the output heatmap is upsampled to match the input size by trilinear interpolation. At inference time, the input clip is center-cropped. For gaze labels, we generate a gaussian kernel centered at the gaze location in each input frame with a kernel size of 19 following Chong et al. (2020). We use a uniform distribution for frames where gaze is not tracked in training and only calculate metrics on frames with fixated gaze in testing as in the work of Li et al. (2018). For the EGTEA Gaze+ (Li et al., 2018) dataset, we determine which frames to calculate metrics on by using the provided label of gaze fixations and saccades. On the Ego4D (Grauman et al., 2022) dataset, no label of gaze type is available. We calculate the euclidean spatial distance of gaze between adjacent frames and consider the tracked gaze to be a saccade if the distance is above a threshold, and treat it as fixation otherwise. We adopt an empirical threshold of 40.

In terms of gaze saccade/fixation prediction, we adopt the same data processing settings as gaze estimation. We aggregate the frame-level labels of gaze type to get the label for each video segment. Specifically, the percentages of saccade frames account for 27% and 17% on EGTEA Gaze+ and Ego4D, respectively. The video segment is labeled as saccade if any sampled frame is annotated as saccade. Otherwise, it’s labeled as gaze fixation. Consequently, the ratio of saccade and fixation is 4:1 on EGTEA Gaze+ and 2:1 on Ego4D.

As for egocentric action recognition, we only implement experiments on EGTEA Gaze+ because Ego4D doesn’t provide action labels. The data processing procedures are identical to gaze estimation except that we set the input dimensions as $8\times 224\times 224$ during training. In testing, the input dimension is $8\times 256\times 256$ following Li et al. (2021). We also adopt more data augmentation including MixUp (Zhang et al., 2017), color jittering and random erasing.

**Table 2:** Comparison with previous methods on EGTEA Gaze+

Methods	F1	Recall	Precision
Center Prior	10.7	32.0	6.4
GBVS (Harel et al., 2006)	15.7	45.1	9.5
EgoGaze (Li et al., 2013)	16.3	16.3	16.3
SimpleGaze	31.3	41.8	16.1
Deep Gaze (Zhang et al., 2017)	34.5	43.1	28.7
Gaze MLE (Li et al., 2021)	26.6	35.7	21.3
Joint Learning (Li et al., 2021)	34.0	42.7	28.3
Attention Transition (Huang et al., 2018)	37.2	51.9	29.0
I3D-R50 (Feichtenhofer et al., 2019)	40.9	57.2	31.8
MotionFormer (Patrick et al., 2021)	42.1	56.4	33.7
MViT (Fan et al., 2021)	43.0	57.8	34.2
GLC-MotionFormer	43.2	59.0	34.1
GLC-MViT	**44**.**8**	**61**.**2**	**35**.**3**

Our complete model is highlighted. The proposed model outperforms previous approaches by a significant margin. See Sect. 4.2.3 for more details

### Experimental Results on Egocentric Gaze Estimation

#### The Design Choice of Global Visual Embedding

We introduce four global context embedding strategies in Sect. 3.1 and Fig. 4. We investigate the performance of these strategies on MViT model (Fan et al., 2021). As shown in Table 1, all four global embedding strategies improve the performance of vanilla MViT model on both the EGTEA dataset and the Ego4D dataset. This result supports our claim that global context is essential for gaze estimation. Among the four embedding strategies, (d) achieves the largest performance improvement on both datasets (+0.9% on EGTEA and +0.8% on Ego4D). This indicates that convolutional layers and the embedded local tokens can facilitate the learning of global context. Thus, we use this strategy in the following experiments. Note that all baseline methods use the same transformer decoder.

#### Evaluation of Global–Local Correlation

We also evaluate the Global–Local Correlation (GLC) module of our model. As presented in Table 1, our full model – MViT+(d)+GLC outperforms the baseline MViT by +1.8% on EGTEA dataset and +2.2% on Ego4D dataset. Specifically, the GLC module contributes to a performance gain of +0.9% on EGTEA and +1.4% on Ego4D (comparing to MViT+(d)). This result suggests that the GLC can break down the mathematical equivalence of global and local tokens in regular self-attention, thereby “highlighting” the global–local connection in the learned representation.

**Does the Performance Improvement Come from Additional Parameters?** It is possible that the performance of our model benefits from additional parameters in the GLC module. In Table 1, we report the results of another baseline model — MViT+(d)+SA., where we remove the GLC module and add a regular self-attention (SA) layer at the same location. Interestingly, the additional SA layer has minor influence on the overall performance (+0.2% on EGTEA and +0.4% on Ego4D). In contrast, our model outperforms this baseline by +0.7% on EGTEA and +1.0% on Ego4D. This result indicates that the performance boost of our method does not simply come from the additional parameters of GLC. Instead, the explicit modeling of the connection between global and local visual features is the key factor in the performance gain. On the other hand, the regular SA layer includes both global–local correlations and local correlations while the proposed GLC module only calculates global–local correlations. The results suggest that local correlations may dilute the global context and thus limit the performance.

#### Comparison with Previous State-of-the-Art

**Table 3:** Comparison with previous methods on Ego4D

Methods	F1	Recall	Precision
Center Prior	14.9	21.9	11.3
GBVS (Harel et al., 2006)	18.0	47.2	11.1
Attention Transition (Huang et al., 2018)	36.4	47.6	29.5
I3D-R50 (Feichtenhofer et al., 2019)	37.5	52.5	29.2
MotionFormer (Patrick et al., 2021)	38.5	55.0	29.6
MViT (Fan et al., 2021)	40.9	57.4	31.7
GLC-MotionFormer	41.0	56.8	32.0
GLC-MViT	**43**.**1**	**57**.**0**	**34**.**7**

Our complete model is highlighted. The model shows consistent superiority over other state of the arts on all metrics. See Sect. 4.2.3 for more details

In addition to these studies to evaluate the components of our model, we compare our approach with prior works. Apart from MViT (Fan et al., 2021), we also plug the global embedding and GLC modules in another transformer-based architecture – MotionFormer (Patrick et al., 2021). Results are presented in Table 2 and Table 3. Note that, for Attention Transition (Huang et al., 2018), I3D-R50 (Feichtenhofer et al., 2019), MotionFormer (Patrick et al., 2021) and MViT (Fan et al., 2021) from Table 2 and all baselines from Table 3, we initialize the model parameters using pretrained checkpoints from Kinetics (Kay et al., 2017) and finetune the models using the same training set as our method. Interestingly, the baseline MViT and MotionFormer easily outperform all previous works that use CNN-based architectures on both the EGTEA dataset and the Ego4D dataset. In addition, our method implemented on MotionFormer (GLC-MotionFormer) outperforms the best CNN model by +2.3% on F1, +1.8% on recall and +2.3% on precision for EGTEA, and +3.5% on F1, +4.3% on recall and +2.8% on precision for Ego4D. The improvement is more prominent with MViT as backbone (GLC-MViT). It surpasses the best CNN model by +3.9% on F1, +4.0% on recall and +3.5% on precision for EGTEA, and +5.6% on F1, +4.5% on recall and +5.5% on precision for Ego4D. These results demonstrate the superiority of using a transformer-based architecture for egocentric gaze estimation as well as the effectiveness and robustness of our proposed method.

We can also observe MotionFormer lags behind MViT by a large margin. This is because MotionFormer directly downsamples the spatial resolution of video frames by 16 in the visual token embedding module, while MViT adopts a multi-scale downsample strategy. The aggressive reduction in spatial dimension keeps high-level semantic information but loses low-level spatial features. Nonetheless, our method can still boost the performance of MotionFormer prominently on the two datasets (+1.1% on EGTEA and +2.5% on Ego4D). It suggests the proposed method can work as an easy-to-use plug-in for other transformer-based models and brings notable gains.

**Fig. 5:**
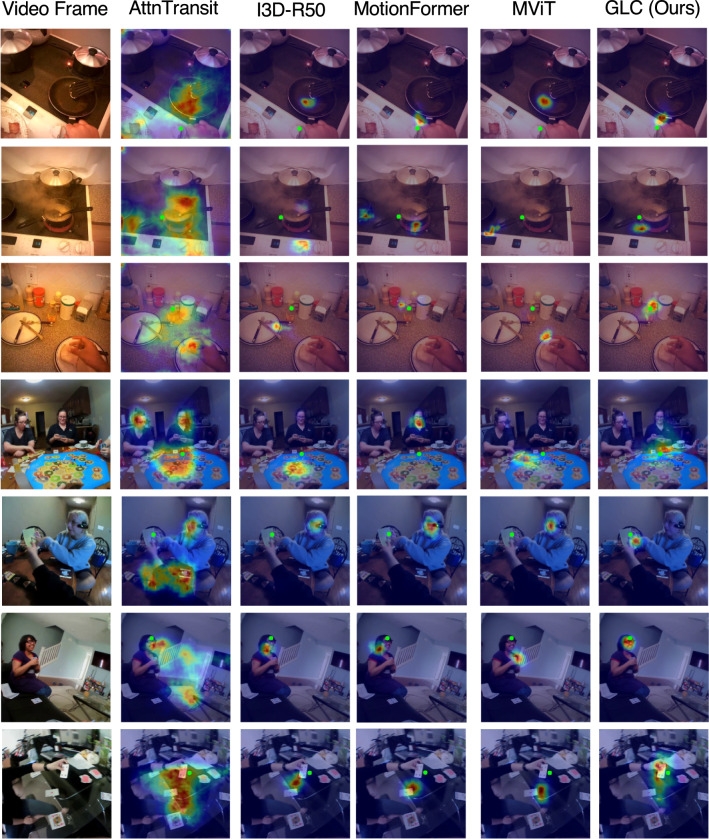
Visualization of gaze estimation. Estimated gaze is represented as a heatmap overlayed on input frames. Green dots denote the ground truth gaze location

**Fig. 6:**
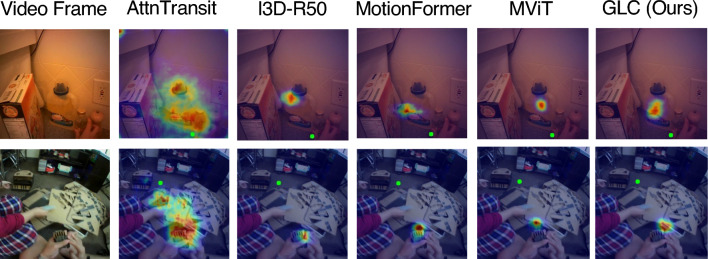
Failure cases for egocentric gaze estimation. Estimated gaze is represented as a heatmap overlayed on input frames. Green dots denote the ground truth gaze location

**Fig. 7:**
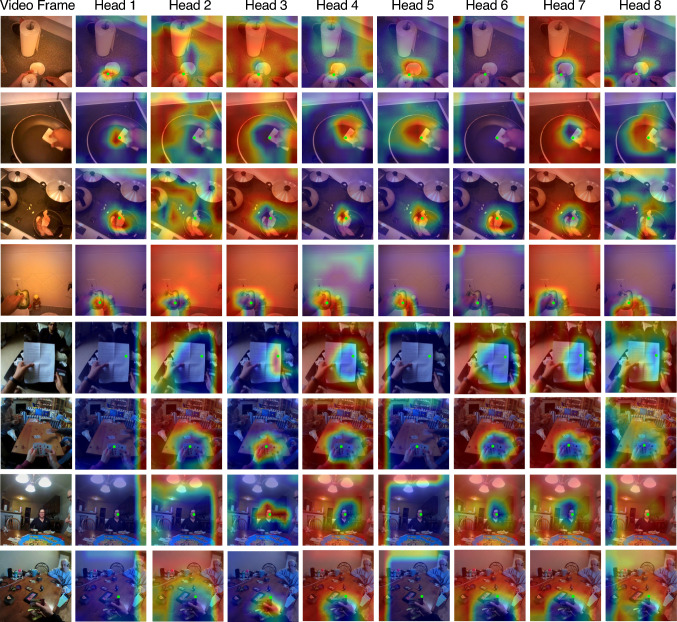
Visualization of the eight heads in global–local correlation module for egocentric gaze estimation. The first four samples are from EGTEA Gaze+ and the last four are from Ego4D. Green dots denote gaze location

Moreover, We note that the improvement of our model is more prominent on Ego4D than EGTEA. We speculate that this is because the Ego4D videos with gaze tracking data are captured under social interaction scenarios that contain interactions with both people and objects, and thus require the model to more heavily consider the global–local connections (e.g. the visual information about a social partner’s gesture to an object) to predict the gaze. Another possible reason is that the Ego4D dataset has more samples to train the transformer-based model.

#### Remarks

**Visualization of Predictions.** We visualize predictions of our model and other previous methods in Fig. 5. Attention transition (Huang et al., 2018) tends to overestimate gaze area which includes more uncertainty and ambiguity. I3D-R50 (Feichtenhofer et al., 2019), vanilla MotionFormer (Patrick et al., 2021) and vanilla MViT (Fan et al., 2021) architectures run into failure modes when there are multiple objects and people in the scene. In contrast, our model, by explicitly modeling the connection between the global and local visual tokens, more robustly predicts the egocentric gaze distribution from the input video clip. We also illustrate examples of failure cases of our model in Fig. 6. Predicting gaze target near the boundary of the frame or in a scene without enough evidence to infer the gaze target remains a challenging problem for our model as well as prior methods.

**What has been learned by the Global–Local Correlation module?** We additionally empirically analyze our proposed GLC module. We first calculate the correlation of the global token and each local token, and then normalize the calculated weights into a probabilistic distribution. A higher score suggests that the GLC captures a stronger connection between the particular local token and the global context. We reshape and upsample these weight distributions to form a heatmap, which we overlay with the original input. Since the GLC module applies a multi-head operation, we visualize the results from different heads in Fig. 7. Interestingly, the correlations captured by the GLC heads are quite diverse. Specifically, on the EGTEA dataset, the maps produced by heads 1, 4, 5, and 8 highlight pixels around the gaze point with different uncertainty (which is illustrated by the size of highlighted area). The other four heads focus on surrounding objects and leave gaze areas unattended. As for the Ego4D data, only head 3 captures the wearers’ attention, while the other heads fully focus on the backgrounds in different aspects. This suggests that our GLC module does learn to model human attention by setting different weights from local to global tokens, capturing many facets of scene information (both around the gaze target and in the background) in the multi-headed attention mechanism. Another important finding is that some heads learn to attend to background pixels to prevent the model from omitting important scene context.

### Experimental Results on Gaze Saccade/Fixation Prediction

Apart from gaze estimation, we demonstrate the capability of our model in capturing the feature of gaze fixation and saccade. We use the same backbone as egocentric gaze estimation but replace the decoder with a linear layer for binary classification. Binary cross-entropy loss is adopted in training and the loss weight for saccade and fixation is set as 1:2 to balance the two categories. Results are presented in Table 4.

Vanilla MotionFormer and MViT can both capture the feature of gaze movement on the two datasets. After embedding the global context into a global token, MotionFormer is boosted by +0.6% on EGTEA and +0.6% on Ego4D while MViT is boosted by +0.8% on EGTEA and +1.1% on Ego4D in terms of average F1. The GLC module further improves the performance by a notable margin. Consequently, our method leads to an overall improvement over the MotionFormer baseline by +1.1% on average F1, +1.1% on mean class accuracy and +0.3% on accuracy for EGTEA as well as +1.8% on average F1, +0.9% on mean class accuracy and +2.5% on accuracy for Ego4D. Furthermore, it also improves MViT by +1.6% on average F1, +1.3% on mean class accuracy, +1.7% on accuracy for EGTEA, as well as +3.7% on average F1, +4.4% on mean class accuracy, +3.2% on accuracy for Ego4D. Obviously, gains on Ego4D are more prominent than EGTEA which is consistent with the phenomenon we observed in gaze estimation and can be explained with the similar reasons (see Sect. 4.2.3). We also note that the improvement of MotionFormer is much smaller than MViT. The possible reason is that MotionFormer calculates correlations along the trajectory of each pixel which might ignore some indicators for saccade (e.g. blurry background). The overall improvements further validate the capability of our method in egocentric gaze behavior modeling.

**Table 4:** Results of gaze saccade/fixation prediction on EGTEA Gaze+ and Ego4D datasets

Methods	EGTEA Gaze+			Ego4D		
	Avg F1	Mean Acc	Acc	Avg F1	Mean Acc	Acc
MotionFormer (Patrick et al., 2021)	56.9	56.5	75.3	59.3	61.4	60.1
MotionFormer + Global Token	57.5	57.4	73.8	59.9	60.3	62.4
MotionFormer + Global Token + GLC	58.0	57.6	75.6	61.1	62.3	62.6
MViT (Fan et al., 2021)	58.0	57.8	74.4	57.9	58.6	59.8
MViT + Global Token	58.8	58.0	**77**.**4**	59.0	60.1	60.5
MViT + Global Token + GLC	**59**.**6**	**59**.**1**	76.1	**61**.**6**	**63**.**0**	**63**.**0**

Avg F1 denotes average F1 and Mean Acc denotes mean class accuracy. Acc is the regular accuracy metric

**Table 5:** Results of action recognition on EGTEA Gaze+

Methods	Cls Token	Pooling	Top1-Acc	Top5-Acc	Mean Cls Acc
MFormer (Patrick et al., 2021)	$\checkmark$		63.4	85.8	55.3
MFormer (Patrick et al., 2021)		$\checkmark$	64.7	86.5	55.6
MFormer + Global Token	$\checkmark$		63.2	**90**.**1**	53.3
MFormer + Global Token		$\checkmark$	64.6	88.7	57.3
MFormer + Global Token + GLC	$\checkmark$		64.3	89.6	56.4
MFormer + Global Token + GLC		$\checkmark$	**66**.**3**	**90**.**1**	**59**.**0**
MViT (Fan et al., 2021)	$\checkmark$		64.6	89.2	54.0
MViT (Fan et al., 2021)		$\checkmark$	63.5	88.7	55.3
MViT + Global Token	$\checkmark$		64.4	88.7	55.3
MViT + Global Token		$\checkmark$	63.1	88.5	54.2
MViT + Global Token + GLC	$\checkmark$		64.8	88.7	56.8
MViT + Global Token + GLC		$\checkmark$	**65**.**3**	**89**.**1**	**57**.**3**

We implemented two methods for classification–adding an additional class token or using global average pooling. We show the generalization capability of the proposed method on two backbones. The complete models are highlighted

### Experimental Results on Action Recognition

In addition to gaze behavior modeling, we also examine the application of our GLC module to the egocentric video action recognition task, and find that our method performs competitively with methods designed specifically for this task on EGTEA Gaze+. Similar to saccade/fixation prediction, we remove the decoder in the gaze estimation model and keep only the visual token embedding, transformer encoder, and GLC modules. However, we further investigate two different ways to obtain class categories for action recognition: adding a class embedding token at the first layer of transformer, or using pooling across all local tokens to obtain a final embedding. Then a fully-connected layer followed by softmax is used to predict probabilities for each category. We implement both strategies and compare our approaches with previous works in Table 5. We conduct these experiments with two backbones only on EGTEA Gaze+ (Li et al., 2018) using the same split as gaze estimation. Note that the Ego4D (Grauman et al., 2022) social benchmark does not contain action labels.

For vanilla MotionFormer (Patrick et al., 2021) and MViT (Fan et al., 2021), class token embedding performs better than or comparably with the pooling operation. For both strategies, simply adding global embedding to MotionFormer only results in minor gains in the performance. Likewise, adding global embedding to MViT has a minor influence on the overall performance ($-$0.2% on top1 accuracy, $-$0.5% on top5 accuracy and +1.3% on mean class accuracy while using the class token, and $-$0.4%, on top1 accuracy, $-$0.2% on top5 accuracy and $-$1.1% on mean class accuracy while using pooling layer). This result suggests that simply embedding global context into an additional token has minor influence on the action recognition performance.

**Fig. 8:**
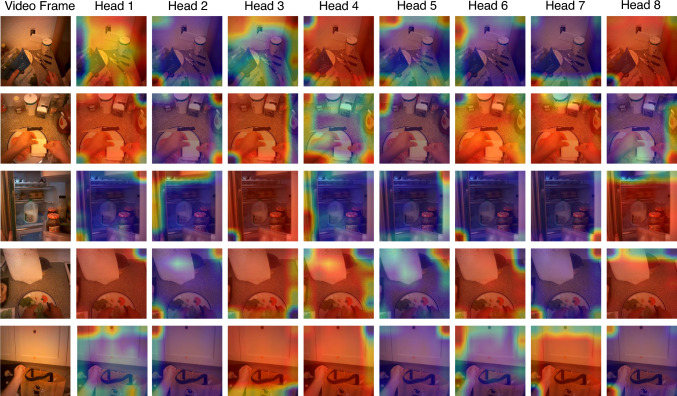
Visualization of the eight heads in global–local correlation module for action recognition on EGTEA Gaze+

In addition, adding our GLC module can only improve the model performance by a small margin when using class token embedding to predict action classes. We hypothesize that this is because only the class token is input into the linear layer for final prediction and re-weighted tokens from GLC are left unused. In contrast, when applying global average pooling on all local tokens, GLC improves top1, top5 and mean class accuracy over the counterpart that doesn’t use GLC (*MotionFormer/MViT+Global Token*) by +1.7%, +1.4% and +1.7% respectively for MotionFormer and +2.2%, +0.6% and +3.1% respectively for MViT. Gains over corresponding MotionFormer baseline are +1.6%, +3.6% and +3.4% on the tree metrics while gains over MViT baseline are +1.8%, +0.4% and +2.0%. These results indicate our proposed GLC module is a robust and general design that also improves the action recognition performance. However, the impact on action recognition is smaller compared with egocentric gaze estimation because our model doesn’t have any specific design for actoin recognition task.

We note that our model achieves a competitive performance for action recognition on EGTEA without additional design for this specific task. Our top1 accuracy of 65.3% exceeds the model from Wang et al. (2020) by +2.2%, and is only a $-$0.2% difference from the recent state-of-the-art method (Hao et al., 2022) for this benchmark of 66.5%. We also want to emphasize that we conduct these action recognition experiments to demonstrate the generalization capability of our proposed GLC module rather than aim to produce SOTA results on action recognition.

Additionally, we visualize the global–local correlation weights of the GLC in Fig. 8. Importantly, the learned global–local correlation is vastly different from the gaze distribution when the model is trained for action recognition; in contrast, a stronger connection between the learned global–local correlation and gaze distribution can be observed when the model is trained for gaze estimation (see Fig. 7). How to design a weakly-supervised model for egocentric gaze estimation remains an open question.

## Limitations and Future Work

**Limitations.** Despite the notable gains from global–local correlation, there are still some limitations in our transformer-based method. The model requires larger computational cost, and therefore may not be feasible for on-device computing (e.g. AR/VR). We note that some recent works on network architecture research (Chen et al., 2021) and knowledge distillation(Lin et al., 2022) seek to reduce the computational cost of transformer architecture. These works actually demand a dense model with strong performance as a starting point. Therefore, our work may provide a foundational step for designing light-weight models for the egocentric gaze estimation setting in the future.

**Future Work.** In this paper, we studied the explicit integration of global scene context for egocentric gaze estimation and proposed a novel modeling approach for this problem. We also showe the results of our proposed architecture on gaze saccade/fixation prediction and egocentric action recognition to demonstrate our model’s generalization cability. Our findings also point to several exciting future research directions:

- Our proposed GLC module has the potential to address other video understanding tasks including visual saliency prediction in third-person video, active object detection, and future forecasting. We plan to study the effect of our method on those tasks in our future work.
- Our proposed GLC fails to learn the gaze distribution when the model is trained to predict the action labels. How to design a weakly supervised model for egocentric gaze estimation using action labels is an interesting problem.
- Our transformer-based model requires larger computational cost, and hence may not be applied for on-device computing. We will continue to study how to combine it with some recent works on network architecture research (Chen et al., 2021) and knowledge distillation (Lin et al., 2022) to reduce the computational cost of transformer architecture.

## Conclusion

In this paper, we develop a transformer-based architecture to address the task of estimating the camera wear’s gaze fixation based only on egocentric video frames. Our key insight is that our global visual token embedding strategy, which encodes global visual information into the self-attention mechanism, and our global–local correlation (GLC) module, which explicitly reasons about the connection between global and local visual tokens, facilitate strong representation learning for egocentric gaze estimation. Our experiments on the EGTEA Gaze+ and Ego4D datasets demonstrate the effectiveness of our approach. We additionally apply our method to a novel gaze saccade/fixation prediction task and the traditional action recognition problem. The proposed method can improve the performance prominently which shows its strong generalization capability. We also implement the global token embedding strategy and GLC module in two backbones to show it can serve as an easy-to-use plug-in to other transformer-based architecture. We believe our work serves as an essential step in analyzing gaze behavior from egocentric videos and provides valuable insight into learning video representations with transformer architectures.

## Data Availability

The data used in the experiments are publicly available online. EGTEA Gaze+ dataset is available via https://cbs.ic.gatech.edu/fpv/. Ego4D dataset is available via https://ego4d-data.org/.

## Code Availability

Codes and train/test split for Ego4D are available on https://bolinlai.github.io/GLC-EgoGazeEst/.
